# SERPINA1 mRNA as a Treatment for Alpha-1 Antitrypsin Deficiency

**DOI:** 10.1155/2018/8247935

**Published:** 2018-06-13

**Authors:** Brendan Connolly, Cleo Isaacs, Lei Cheng, Kirtika H. Asrani, Romesh R. Subramanian

**Affiliations:** Alexion Pharmaceuticals Inc., 75 Sidney St, Cambridge, MA 02139, USA

## Abstract

Alpha-1-antitrypsin (AAT) deficiency is a genetic disorder that produces inactive/defective AAT due to mutations in the SERPINA1 gene encoding AAT. This disease is associated with decreased activity of AAT in the lungs and deposition of excessive defective AAT protein in the liver. Currently there is no specific treatment for liver disease associated with AAT deficiency. AAT lung disease is often treated with one of several serum protein replacement products; however, long-term studies of the effectiveness of SerpinA1 replacement therapy are not available, and it does not reduce liver damage in AAT deficiency. mRNA therapy could potentially target both the liver and lungs of AAT deficient patients. AAT patient fibroblasts and AAT patient fibroblast-derived hepatocytes were transfected with SERPINA1-encoding mRNA and cell culture media were tested for SerpinA1 expression. Our data demonstrates increased SerpinA1 protein in culture media from treated AAT patient fibroblasts and AAT patient fibroblast-derived hepatocytes. In vivo studies in wild type mice demonstrate SERPINA1 mRNA biodistribution in liver and lungs, as well as SerpinA1 protein expression in these two target organs which are critically affected in AAT deficiency. Taken together, our data suggests that SerpinA1 mRNA therapy has the potential to benefit patients suffering from AAT deficiency.

## 1. Introduction

Alpha-1-antitrypsin deficiency (AATD) is a devastating disease with a lack of adequate therapies. Current therapies only protect the lungs and in advanced cases require frequent intravenous injections of plasma-derived AAT protein. Patients with AATD are predisposed to obstructive pulmonary disease and liver disease (e.g., cirrhosis and hepatocellular carcinoma in children and adults) [1–3]. Approximately 1–5% of patients with diagnosed chronic obstructive pulmonary disease (COPD) are estimated to have alpha-1-antitrypsin deficiency [4]. While extremely rare, emphysema in children with AATD has been reported [3] and the incidence of liver disease increases with age [3]. Severe symptoms occur later in life, particularly in smokers. Hepatic symptoms of AATD are seen late in life and often require liver transplantation. AATD is an inherited disease caused by mutations within the* SERPINA1* gene [5]. The SERPINA1 gene has two alleles designated as M alleles (normal patients have an MM genotype). The most common allele resulting in severe AAT deficiency is called Z (E342K) [6, 7]. It has been reported that 95% of severe AATD patients have a genotype of ZZ and have only 10–20% of SerpinA1 serum levels [6]. In cases of ZZ mutation the SerpinA1 protein cannot fold properly and will not be secreted by hepatocytes; therefore AATD is not caused by an inability to make protein but an inability to secrete functional protein. The accumulation of ZZ SerpinA1 in hepatocytes results in severe liver damage [6]. Homozygous PIZZ is quite common and occurs in 1:2500-3000 births in North America and Europe.

Alpha-1-antitrypsin (AAT), also known as SERPINA1 (serine protease inhibitor, group A, member 1), is a secreted protein produced mainly by hepatocytes and to a smaller extent by mononuclear phagocytes, neutrophils, and airway/intestinal epithelial cells [5, 8]. SerpinA1 is a circulating glycoprotein which is also water soluble and tissue diffusible [5]. The primary role of SerpinA1 is serine protease regulation and the main site of this action is in the lungs. There, SerpinA1 binds and inactivates neutrophil elastase thereby protecting alveolar tissues from proteolytic degradation during inflammatory responses [9]. SerpinA1 is the most abundant circulating anti-protease and normal plasma concentrations of SerpinA1 usually reach 1-2g/L [5].

AATD is characterized by polymerization of misfolded SerpinA1 proteins in the rough endoplasmic reticulum of hepatocytes which decreases concentration and activity of SerpinA1 in blood and tissues [6]. For patients with severe deficiency, SerpinA1 serum levels are below 50 mg/dL while normal levels are at 200mg/dL. With low levels of circulating SerpinA1, the lungs are not protected from neutrophil elastase, an enzyme that destroys alveoli and causes lung disease. Therefore, the most frequent clinical complications of AATD are pulmonary emphysema and liver disease [10].

To date only AAT augmentation therapy is available for treatment of AATD. AAT augmentation therapy was developed in 1987 and is only currently approved for commercial use in selected patients with severe AATD related pulmonary emphysema [6, 11]. AAT augmentation only helps to supply SerpinA1 to the lungs where it can help prevent neutrophil elastase damage. Since serum deficiency is not the cause for liver damage there is no specific treatment for patients suffering from liver disease associated with AATD. The only treatment is supportive care for typical liver failure and liver transplant for patients with decompensated cirrhosis [12].

AATD is a monogenic disorder which makes it an ideal target for mRNA therapy. mRNA is a novel modality that is potentially applicable for a wide range of diseases because of its enormous flexibility over traditional biologic and enzyme replacement therapeutics. mRNA-based therapeutics utilize the host cell's endogenous machinery for the production and posttranslational modification of target proteins, allowing for the development of therapeutics for diseases not previously targetable. We carried out in vitro and in vivo studies to determine if we could express exogenously encoded SerpinA1 and target the mRNA/protein to the appropriate organ.

mRNA therapy could potentially target both the liver and lungs of AAT deficient patients (pending current experiments). Our data demonstrated increased SerpinA1 protein in cell culture media of treated AAT patient fibroblasts, as well as in the cell culture media of AAT patient fibroblast-derived primary hepatocytes. Moreover, in WT animals receiving SERPINA1 mRNA via intravenous injection, both SERPINA1 mRNA and SerpinA1 protein were observed to be localized in both the liver and lungs. Taken together, our data suggests that mRNA-mediated expression of SerpinA1 protein in lung could inhibit neutrophil elastase, while it could promote MM isoform expression in liver and provide multiple routes of benefit for AAT patients.

## 2. Materials and Methods

### 2.1. AATD Patient Fibroblasts

*Cell Culture Conditions*. The following patient fibroblasts were obtained from the Coriell Institute for Medical Research, Cell Repository (Camden, NJ): GM11423 and GM12445 from AATD patient liver and GM02522 from AATD patient skin and the normal control fibroblast line ND34769 from skin. Cells were grown in Eagle's Minimum Essential Medium with Earle's salts and nonessential amino acids (Thermo Fisher Scientific, Inc., Waltham, MA) supplemented with 15% fetal bovine serum and incubated at 37°C in 5% CO_2_.

*Transfection Conditions*. Patient fibroblasts were transfected with 2.5ug of each mRNA and 4 *μ*l of mRNA-In lipid transfection reagent (MTI-GlobalStem, Gaithersburg, MD), in 3 independent experiments with 3 biological replicates per experiment. Each mRNA was diluted to 2.5 *μ*g in OptiMEM (Thermo Fisher Scientific, Inc., Waltham, MA) and similarly appropriate volume of mRNA-In was diluted in OptiMEM per well and protocol was followed according to manufacturer's recommendation. Diluted mRNA was mixed with diluted lipid and allowed to incubate for 15 minutes at room temperature before adding to cells. After addition of mRNA-lipid mix to patient fibroblasts, cells were returned to incubator at 37°C with 5% CO_2_. After 24 hours cells were gently washed with PBS twice and lysed in fresh RIPA buffer (Sigma-Aldrich, St. Louis, MO) with 1X protease inhibitors (Sigma-Aldrich, St. Louis, MO).

Cell lysis and total protein quantification: The patient fibroblast cell lysates were collected in 0.5mL tubes (Eppendorf, Hamburg, Germany) and centrifuged at 18000 x g at 4°C to remove cell debris. After centrifugation, clear supernatant was carefully transferred to a separate labelled tube and placed on ice. Total protein concentration was determined with BCA assay kit (Thermo Fisher Scientific, Inc., Waltham, MA) in duplicate in 96-well format according to manufacturer's protocol.

*Wes Automated Capillary Electrophoresis for SERPINA1 Expression*. The final samples of 5 *μ*l each at 0.4 mg/mL protein concentration were prepared following Protein Simple Wes manufacturer's instructions. Lysates were analyzed using the Protein Simple Wes size-based capillary electrophoresis system (ProteinSimple Wes; ProteinSimple, San Jose, CA). The size-separated proteins were probed with antibodies specific for SERPINA1 at 1:125 dilution (Novus NBP1-90309, Novus Biologics, Littleton, CO) and the housekeeper Thioredoxin at 1:1000 (CST 2429, Cell Signaling Technologies Inc., Danvers, MA), visualized using labelled rabbit secondary antibodies and quantitated using the manufacturers' software. For each lane, the area under the curve (AUC) of the SERPINA1 was normalized to the AUC of the Thioredoxin control.

### 2.2. Alpha-1 Antitrypsin Deficient Hepatocytes

*Cell Culture Conditions*. AATD patient-derived hepatocytes were generated from AATD fibroblasts by DefiniGen (Cambridge UK) using a proprietary differentiation protocol. Cells were grown in DTM media and DRM media (DefiniGen, Cambridge UK) and plated at 500,000 cells per well in a 24-well plate. Cells were grown in in hypoxic conditions at 37°C in 5% CO_2_ and 5% O_2_ with the media being changed every two days for 10 days prior to transfection based on proprietary manufacturer's protocol (DefiniGen, Cambridge UK). Cells were grown in hypoxic conditions per manufacturer's instructions, which enabled cell growth, while nonhypoxic conditions reduced cell growth.

*Transfection Conditions and Protein Expression by ELISA*. AATD Hepatocytes were transfected with 4ul of Lipofectamine® 2000 (Thermo Fisher Scientific, Inc., Waltham, MA) per 2.5ug mRNA, in 3 independent experiments with 3 biological replicates per experiment. Cells were then incubated for 24, 48, and 72 hours before the cell culture media were removed and assayed for SerpinA1 protein concentration using an ELISA from Abcam (Cambridge, MA) and following manufacturer's protocol.

### 2.3. *In Vivo* Studies

All experiments, animal housing, handling, and experimental procedures/protocols were approved and performed in accordance with Alexion Pharmaceuticals Inc. IACUC guidelines and regulations. C57BL/6 male mice (6-week-old) were used in this study. Mice were kept in a temperature-controlled environment with a 12-h/12-h light-dark cycle, with a standard diet and water ad libitum.

*Formulation of mRNA into Lipid Nanoparticles and In Vivo Dosing*. mRNA constructs were formulated using a self-assembling ionizable lipid nanoparticle (LNP) containing SERPINA1 mRNA. A GFP encoding mRNA was used as an mRNA-loaded vehicle control and PBS was used as a negative control in this experiment. LNPs were prepared by rapid mixing of mRNA in acetate buffer at pH 4.0 with a lipid mix, Hepato9 mRNA kit (Precision NanoSystems), suspended in ethanol at a lipid-to-drug ratio of ~11 (wt/wt). Animals were injected with a single intravenous dose of 1.5 mg/kg of formulated mRNA through the tail vein. Animals were sacrificed 24 hours after injection and tissues were fixed and processed for in situ hybridization (ISH) and immunohistochemistry (IHC).

*Sample Collection and FFPE Sample Preparation*. Animals were necropsied, and the left lateral lobe was placed flat in tissue cassettes (Fisher Scientific). Cassettes were then placed into a container filled with 10% Neutral Buffered Formalin, NBF (Sigma) fixative solution at minimum 1:20 ratio of tissue volume versus the fixative solution. Samples were allowed to fix for a minimum of 24h but no more than 48h at room temperature. After tissue fixing, the cassette was placed into a container filled with PBS for not more than 3 days. After PBS wash, liver tissues were processed and embedded into paraffin blocks.

### 2.4. Immunohistochemistry and In Situ Hybridization Procedures

Immunohistochemistry for SERPINA1 was performed on formalin-fixed paraffin-embedded, 5 *μ*m tissue sections using an antibody to SERPINA1 (Atlas, HPA001292 at 1:100). Immunostaining was performed on the Leica BOND RX platform (Leica Biosystems, Leica Microsystems Inc., Buffalo Grove, IL) according to standard protocols with appropriate positive controls. Antigen retrieval, standard on the Leica BOND RX platform, utilized the Leica ER1, EDTA-Tris, pH 8.0 solution. Slides were dehydrated in a graded ethanol series, cleared in xylene, and mounted using Cytoseal. Slides were imaged using the VS120 system at 40x magnification (Olympus).

For mRNA visualization, in situ hybridization (ISH) was done. Custom RNA in situ hybridization probe (Affymetrix, Santa Clara, CA) was designed to detect the SERPINA1 mRNA. Automated RNA ISH assay was performed using the ViewRNA eZ-L Detection Kit (Affymetrix, Santa Clara, CA) and the Leica BOND RX platform (Leica Biosystems, Leica Microsystems Inc., Buffalo Grove, IL) was used. Formalin-fixed, paraffin-embedded (FFPE) tissues were prepared at 5 um thickness on glass slides and put on the Leica BOND RX. The ViewRNA eZ-L assay was run according to manufactures protocol but, briefly, RNA was unmasked with a 10-min incubation at 95°C in Bond Epitope Retrieval Solution 1 (Leica Biosystems, Buffalo Grove, IL), followed by 20-min incubation with Proteinase K from the Bond Enzyme Pretreatment Kit at 1:1000 dilution (Leica Biosystems, Buffalo Grove, IL). Hybridization was performed with SERPINA1 1:40 probe dilution followed by FastRed detection. To ensure RNA integrity and assay procedure, additional sections were also hybridized with a probe for peptidyl prolyl isomerase B (PPIB), an endogenous housekeeping protein used as a positive control for ISH. To control for nonspecific staining, control probe against bacterial protein dihydrodipicolinate reductase (dapB) was used as a negative control. All probes were at a 1:40 dilution. Slides were air dried, cleared in xylene, and mounted using Cytoseal. Slides were imaged using the VS120 system at 40x magnification (Olympus).

## 3. Results

### 3.1. mRNA-Derived Human SerpinA1 Expression in Cell Lysates and Cell Media of Patient Fibroblasts and Patient Fibroblast-Derived Primary Hepatocytes

**Figure 1:**
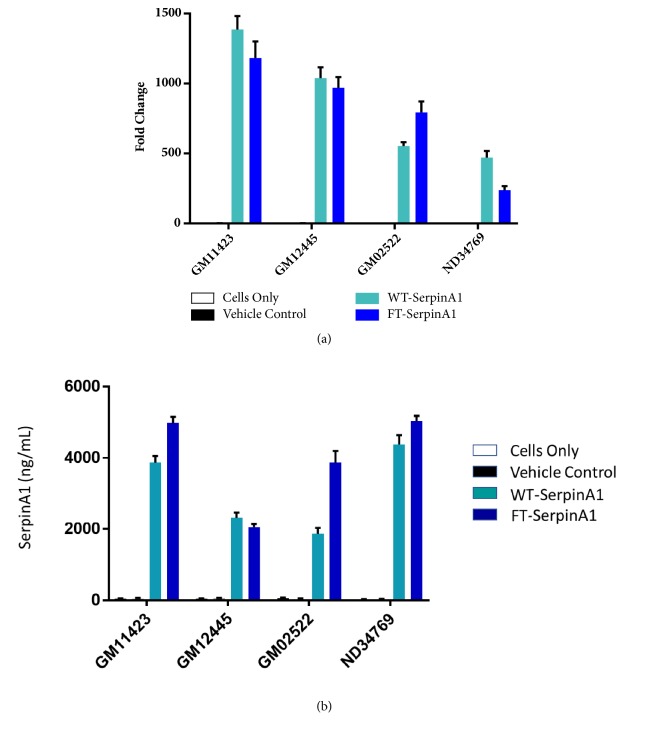
In vitro human SerpinA1 expression in cell lysates (a) and cell media (b) from AATD patient fibroblasts and normal skin fibroblasts. Vehicle control is GFP mRNA; WT-SERPINA1 is WT mRNA sequence; FT-SerpinA1 is flag-tagged mRNA sequence.

Human SERPINA1 mRNA from TriLink was transfected into AATD patient fibroblasts, obtained from the Coriell Institute. Significant increase in human SerpinA1 protein expression was observed in cell lysates (Figure 1(a)) and cell culture media (Figure 1(b)) upon transfection of either wild type (WT) or FLAG-tagged (FT) SerpinA1.

**Figure 2:**
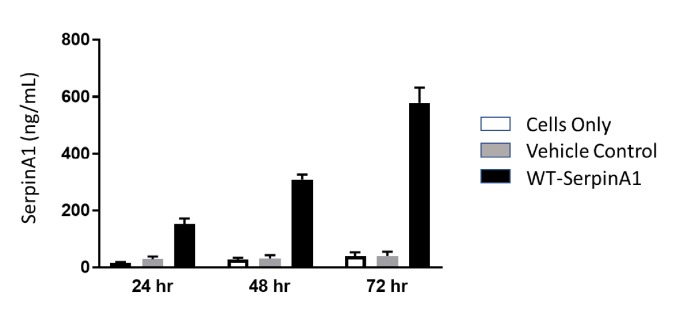
In vitro human SerpinA1 expression in cell culture media obtained from patient fibroblast-derived hepatocytes (DefiniGen, Cambridge UK).

Human SERPINA1 mRNA transfection was also carried out in patient fibroblast-derived primary hepatocytes obtained from DefiniGen. Secretion of WT SerpinA1 protein into the culture medium was increased at 24h compared to the vehicle only GFP mRNA control. We observed a consistent increase in SerpinA1 protein expression over time as seen at 48h and 72h after transfection (Figure 2).

### 3.2. In Vivo Human SerpinA1 Expression in WT Mice

**Figure 3:**
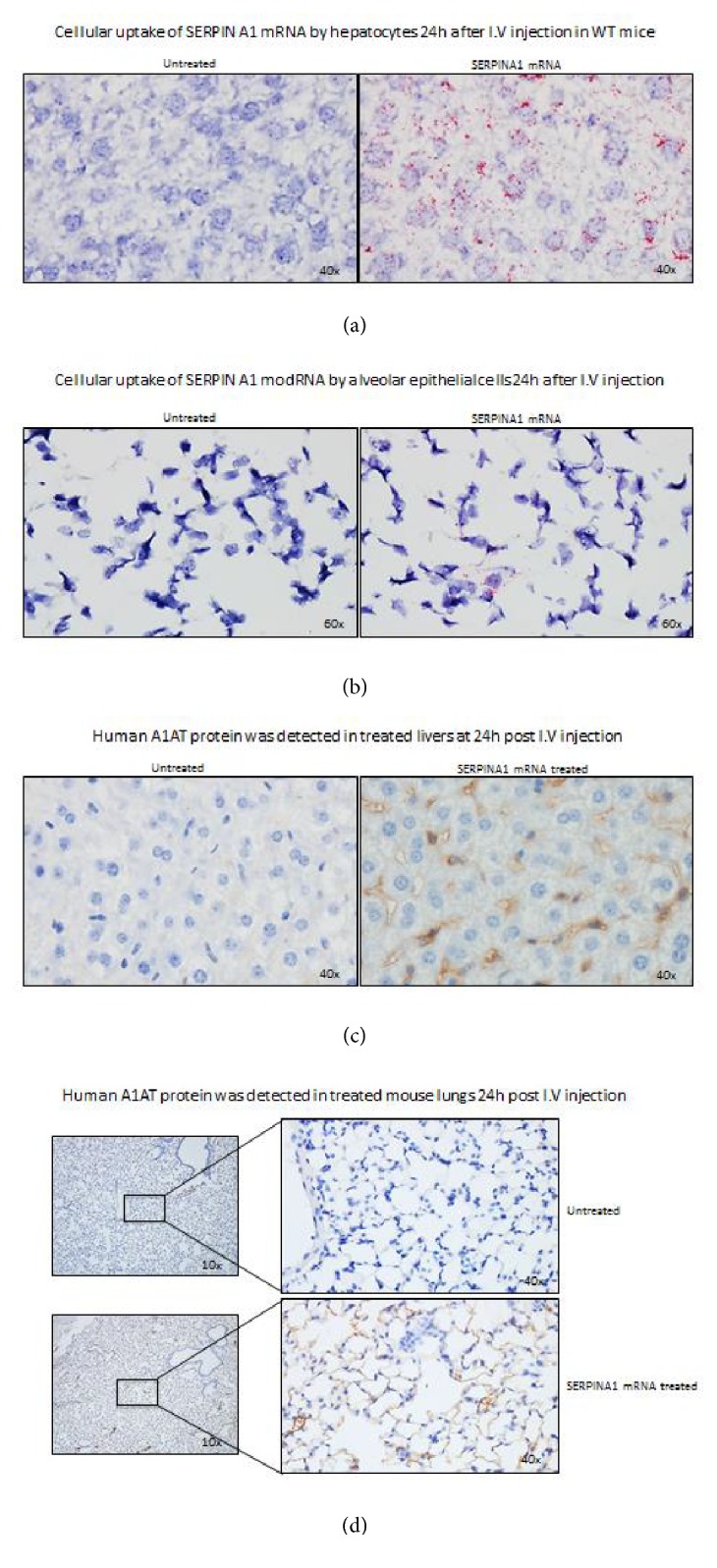
In vivo human SERPINA1 mRNA biodistribution in liver (a) and the lungs (b) of WT mice 24h after i.v. injection was detected by ISH. Red dots in the image represent mRNA. SerpinA1 protein was visualized in liver (c) and lungs (d) by IHC.

Human SerpinA1 protein expression was subsequently evaluated in WT C57bl/6 mice. Human SERPINA1 mRNA was observed in liver (Figure 3(a)) as may be expected given that the mRNA is encapsulated in a lipid nanoparticle which generally delivers a large amount of its cargo to the liver. An intriguing finding was that SERPINA1 mRNA was also detected in the lungs of mice (Figure 3(b)). SerpinA1 protein expression was detected using a human specific SerpinA1 antibody. SerpinA1 protein was seen in both liver (Figure 3(c)) and the lungs (Figure 3(d)). The sinusoidal distribution pattern of SerpinA1 protein expression in the liver indicates that SerpinA1 protein is probably being secreted into the blood by hepatocytes.

## 4. Discussion

AATD is a devastating disease with a need for efficient therapies to benefit patients. Current augmentation therapy is inadequate to avoid long-term damage to the liver of AATD patients. Currently many groups are pursuing novel modalities to provide much needed therapies such as gene therapy. For example, bone marrow-derived stem cell transplantation has been tested in AATD mice and demonstrated some rescue of hepatocyte deterioration [13]. Gene replacement strategies are also being tested in the clinic [14, 15], wherein a WT SERPINA1 gene is inserted into the liver and expresses the appropriate SerpinA1 protein. Current methods still need to be optimized and AAV-mediated gene delivery can cause antibody-mediated decrease in protein secretion or an immunologic reaction to the viral capsid.

We evaluated the effect of transient expression of SERPINA1 mRNA in mouse liver as mRNA delivery to liver is relatively straightforward and established via lipid nanoparticles (LNP), and we can administer LNP-encapsulated mRNA without significant immune reactions. Initially we tested our hypothesis in AATD patient fibroblasts and patient fibroblast-derived primary hepatocytes. Our data clearly demonstrates that we can encode SERPINA1 with mRNA and subsequently express SerpinA1 protein from both cell types. We also demonstrate that SerpinA1 protein is secreted from these cells and protein levels can be measured in the supernatant/cell culture medium. This implies that the mRNA-encoded SerpinA1 protein is folded and modified appropriately within the cell to enable secretion out of the ER and into cell culture medium which is a key feature of this mRNA therapy. Utilizing mRNA therapy, we can utilize the body's own cells as a factory to synthesize protein, posttranslationally modify it, and finally secrete it into the blood stream. Preliminary analysis of cell culture medium indicates that the secreted SerpinA1 is enzymatically active in a neutrophil elastase inhibition assay (data not shown). Our data in Figures 1, 2, and 3 suggest that levels of SerpinA1 protein are significantly higher than baseline or negative control levels. These levels should be sufficient to decrease neutrophil elastase activity in plasma and the lungs and thereby prevent alveolar degradation and emphysema, which are characteristic of AATD.

We extended our studies to determine if mRNA-encoded SERPINA1 could be expressed in vivo. Our studies demonstrate that intravenously injected human SERPINA1 mRNA reached both the liver and lungs in WT mice (Figures 3(a) and 3(b)). Thus, lipid-encapsulated mRNA therapy could potentially target both sites of AATD (liver and lung). More importantly, human SerpinA1 protein was produced in both the liver and lungs of WT mice (Figures 3(c) and 3(d)). The SerpinA1 protein expression pattern in the liver sinusoids indicates that proteins are being secreted by the hepatocytes or another liver cell type such as stellate cells that normally express SERPINA1. Our data cannot rule out the possibility that the mRNA enters or is expressed by other hepatic cells besides hepatocytes, and this will need further work. It is possible that human SerpinA1 protein in the lungs could be coming from two sources: the SERPINA1 mRNA that reached the lungs via i.v. injection, as well as the secreted human SerpinA1 produced by human SERPINA1 mRNA in the liver. In either case, the potential for SerpinA1 protein expression and localization in the lungs and liver is promising as it could block neutrophil elastase-mediated damage in the lungs while possibly decreasing PIZZ forms in the liver. The latter hypothesis is based on the studies from Karadagi et al. [9] wherein an increase in SerpinA1 protein by augmentation therapy demonstrated a decrease in the PIZZ form in AATD patient PBMC. This raises the possibility that mRNA-encoded SerpinA1 protein, when expressed in the liver, could decrease hepatic production of the PIZZ form and subsequently inhibit hepatic fibrosis. Inhibition of hepatic fibrosis and ER stress could ultimately reduce/eliminate the AATD patient's need for a liver transplant. Our hypothesis needs to be tested in vivo in an AATD model that demonstrates liver fibrosis and progression to hepatocellular carcinoma.

While these are exciting data, we would like to point out that mRNA therapies need to be improved to potentially provide chronic benefit for AATD patients. Current half-lives for exogenously delivered mRNA are typically 24–48h in tissue, while protein half-lives vary depending on the protein and its degradative mechanisms. Strategies to increase the half-life of mRNAs are currently under investigation and early data indicate that we can increase the duration of mRNA stability by modulating the UTRs flanking the codon-optimized mRNA [16]. Rational protein engineering strategies are also targeted to facilitate increased protein half-life which results in increased enzymatic activity and ultimately a decreased need for dosing of the mRNA therapy [17]. These novel improvements could result in an mRNA therapy that is dosed infrequently and maintains sufficient enzyme activity, e.g., of SerpinA1 protein, to provide clinical improvement. Another aspect that would improve mRNA therapies is the safety of LNP delivery whereby we can dose LNPs every few weeks. Current LNP technology facilitates a once-a-month dosing regime but does not support a more frequent dosing if we pair dosing with efficacy. As mentioned earlier, LNP-encapsulated mRNA therapies can be redosed, unlike some AAV-gene therapies, and the dose can be titrated to provide maximum patient benefit.

## 5. Conclusion

Our studies present the potential of a new therapeutic modality, mRNA therapy, whereby a missing or nonfunctional protein can be replaced to carry out normal functions. Our findings demonstrate that lipid-encapsulated mRNA can be targeted to the lung and liver which are the two primary sites of disease in AATD. While previous studies exploring mRNA therapy for AATD have shown protein expression at 24h in A549 and HEK293 cells [18], our studies demonstrate SerpinA1 protein expression in AATD patient fibroblasts and patient-derived hepatocytes which are relevant cell lines for the disease. In addition, we extend previous studies in this field by demonstrating that lipid-encapsulated mRNA can deliver to mouse liver and lung and produce SerpinA1 protein to be functionally relevant for patients. Further research needs to be conducted in an AATD disease model and to ensure the safety of multiple dosing of mRNA in rodents and higher animals prior to considering this modality for human use. Taken together, our studies raise the interesting possibility that mRNA therapy utilizing SERPINA1 mRNA could be beneficial for AATD patients.

## Data Availability

All data supporting results published in this article are presented in the article. There are no other data available.
